# P-2095. Ultrasensitive *Clostridioides difficile* Toxin A/B Assay (in Development) for Higher Diagnostic Accuracy

**DOI:** 10.1093/ofid/ofae631.2251

**Published:** 2025-01-29

**Authors:** Gipshu Dave, Tiffany Truong, Mariya Soban, Justin Nguyen, Frank Zaugg, Valerie Brachet, Peter Wagner, Johanna Sandlund

**Affiliations:** Fluxus, Inc., Sunnyvale, California; Fluxus, Inc., Sunnyvale, California; Fluxus, Inc., Sunnyvale, California; Fluxus, Inc., Sunnyvale, California; Fluxus, Inc., Sunnyvale, California; Fluxus, Inc., Sunnyvale, California; Fluxus, Inc., Sunnyvale, California; Fluxus, Inc., Sunnyvale, California

## Abstract

**Background:**

Diagnostic tests for *Clostridioides difficile* infection (CDI) have either poor sensitivity or specificity, which has led to guideline recommendations for complex multistep testing algorithms. Ultrasensitive *C. difficile* toxin A/B measurements correlate with disease and may offer a standalone solution for CDI diagnosis. Fluxus develops ultrasensitive, target-agnostic, and low-complexity single-molecule diagnostic instruments based on optofluidic technology that combines integrated optics and microfluidics on a single chip-based system. Here, we report on the preliminary analytical performance of *C. difficile* toxin A and B assays (in development) powered by the Fluxus technology.

**Figure:**
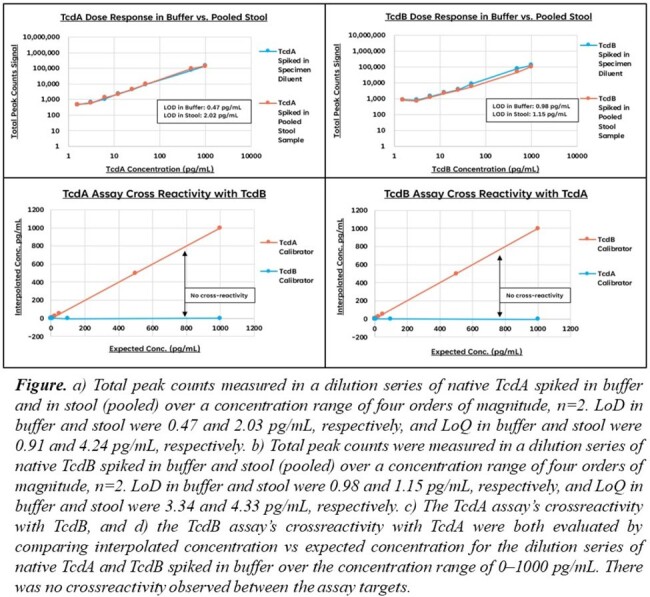

**Methods:**

*C. difficile* toxin A (TcdA) and B (TcdB) were spiked into buffer and stool (pooled) and tested across a wide range of TcdA/TcdB concentrations to give dose-response curves and estimate limits of detection (LoD; assay background +2.5 STD of background) and quantification (LoQ; assay background +10 STD of background). TcdA and TcdB concentrations were calculated from a four-parameter logistic nonlinear regression-based calibration curve. Crossreactivity between the toxins was assessed. Further evaluation of expanded analytical and clinical performance is ongoing.

**Results:**

LoDs in buffer for the prototype TcdA and TcdB assays were 0.47 and 0.98 pg/mL, respectively, and LoQs were 0.91 and 3.34 pg/mL, respectively, with a high dynamic range. LoDs in spiked stool for the TcdA and TcdB assays were 2.02 and 1.15 pg/mL, respectively, and LoQs were 4.25 and 4.35 pg/mL, respectively. There was no crossreactivity between the assay targets across a 4-log dynamic range.

**Conclusion:**

Fluxus’ ultrasensitive *C. difficile* toxin A/B assays in development can detect TcdA and TcdB at pg/mL concentrations. Preliminary evaluation of spiked stool samples indicates a comparable dilution linearity to calibrator dose response in buffer. An ultrasensitive *C. difficile* toxin A/B assay has the potential to provide both high clinical sensitivity and specificity and may replace current testing methods.

**Disclosures:**

Gipshu Dave, MS, Fluxus, Inc.: Stocks/Bonds (Public Company) Tiffany Truong, MS, Fluxus, Inc.: Stocks/Bonds (Public Company) Mariya Soban, MS, Fluxus, Inc.: Stocks/Bonds (Public Company) Justin Nguyen, BS, Fluxus, Inc.: Stocks/Bonds (Public Company) Frank Zaugg, PhD, Fluxus, Inc.: Stocks/Bonds (Public Company) Valerie Brachet, PhD, Fluxus, Inc.: Stocks/Bonds (Public Company) Peter Wagner, PhD, Fluxus, Inc.: Stocks/Bonds (Public Company) Johanna Sandlund, MD, PhD, Fluxus, Inc.: Stocks/Bonds (Public Company)

